# Designing AI for distress: an iterative case study of a hierarchical agent for psychosocial support

**DOI:** 10.3389/frai.2026.1772215

**Published:** 2026-04-24

**Authors:** Vaishnavi Patturajan, Devipreya Ravikumar, Sneghavalli Narayanan, Vallidevi Krishnamurthy, Stephen Santhosh

**Affiliations:** 1 School of Computer Science and Engineering, Vellore Institute of Technology, Chennai, Tamil Nadu, India; 2 DelMag Innovations, Chennai, Tamil Nadu, India

**Keywords:** AI-based guidance, confidentiality, emotional support, escalation, Gemini API, large language model, mental health chatbot, psychosocial assistance

## Abstract

**Introduction:**

In recent years, psychosocial issues like bullying and ragging have risen all around the world. Even though there are some victims who report such cases, most of them never do so because of fear, stigma, or mistrust, mainly among students. This scenario highlights the necessity of safe, anonymous, and easily available support system. The promising approach to solving this problem is conversational AI because it allows people to request assistance in a simple and an easy-going way. However, conversational AI systems often lack contextual sensitivity, emotional intelligence, and appropriate support response mechanisms. To address this, there is a need for structured frameworks integrating human-in-the-loop oversight along with preventive and actionable insights to ensure ethical and proportional responses. This paper introduces SocialWellbeing, a socio-technical system which is designed to overcome psychosocial harms in a three-pillar framework.

**Methods:**

The initial pillar offers artificial intelligence-based first-line care whereby users can share their issues anonymously and get caring responses. The second pillar facilitates human-guided escalation where more serious cases are passed to trusted advisors or authorities to provide real-life support. The third pillar is devoted to data-driven prevention, where anonymized data are used to identify trends and serve as a guide in the institutional preventive efforts.

**Results:**

The AI system was tested in two stages. Phase 1 involved prompt engineering that identified the types of incidents, detected the emotions, and generated supportive responses. Phase 2 improved this strategy with a chain-of-thought strategy, which was A.G.E. (Acknowledge-Guide-Escalate) oriented and explicit guidelines and escalation logic to add reliability and clarity to the strategy. Results of this evaluation show that Phase 2 improved the Gemini 2.5 Flash model's classification accuracy to approximately 88%–89%, compared to 84%–86% in Phase 1, with empathy scores increasing from 4.2–4.3 to 4.4–4.8 on a five-point scale. The level of empathy also went up on a five-point scale to 4.4–4.8 as compared to 4.2–4.3.

**Discussion:**

The findings indicate that a structured, hierarchical approach improves the reliability and safety of psychosocial AI systems. The Acknowledge-Guide-Escalate (A.G.E.) framework enables consistent emotional recognition and contextually relevant guidance. The inclusion of human-in-the-loop oversight and preventive, actionable insights strengthens ethical alignment and response quality.

## 1 Introduction

The issue of the psychosocial wellbeing of members of educational and training institutions has turned into a chronic but under-recognized problem in the world. The UNESCO and the World Health Organization (WHO) reports point to the fact that cases of bullying, harassment, and emotional abuse are still prevalent in the learning settings, which may cause anxiety, depression, and even suicidal feelings (Han, 2025; Thakkar et al., 2021). Although there is formal anti-violence and counseling processes in place, they are usually incapable of reflecting the real extent of the issue due to the persistent underreporting.

It is postulated that non-reporting is often based on the fear of punishment, a fear of image, perceived inefficacy of institutional procedures, and skepticism about confidentiality (Fitzpatrick et al., 2017; Murray-Harvey, 2010). This means that the affected people be they students, trainees and staff members are usually left to suffer in silence. The mental health of individuals is worsened as well as the institutional harmony, productivity, and retention rates are discouraged due to this silent suffering.

In addition to the immediate emotional impact, long-term exposure to unresolved psychosocial damage may have long-term behavioral and cognitive outcomes by impairing the ability of the individuals to participate in teamwork, empathize, and excel in studies (Han, 2025; Murray-Harvey, 2010). These results indicate the need for mental-health services in institutions to become more proactive and technology-intensive, paving the way for artificial intelligence (AI) to augment traditional psychosocial services.

The use of artificial intelligence is an increasing possibility to supplement the traditional psychosocial support framework. The latest developments in conversational agents and affective computing can enable AI systems to re-enact empathy and interact with people in a text-based chat (Inkster et al., 2018; Fitzpatrick et al., 2017). The latter chatbots can be available 24 h a day, perceived as reducing social judgment, and can serve as a harmless point of entry to those unwilling to seek help (Fulmer et al., 2023).

This promise is however hedged with grave moral and technical dangers. Research cautions that AI solutions in the mental-health setting can generate false impressions of knowledge without necessarily empathizing and find it hard to comprehend contextually sensitive confessions and even fail to respond to a crisis appropriately (Pichowicz et al., 2025; Naik et al., 2025). Further, the problem of trust and transparency, as well as data privacy, are still the key challenges (Coghlan et al., 2023; Naik et al., 2025; Weidinger et al., 2021). The researchers have highlighted that AI should be adopted as an auxiliary layer in a psychosocial system governed by humans, and not as an alternative to the human empathy or professional skills (Rubin et al., 2024).

To address the challenges raised, the SocialWellbeing framework highlights that a three-pillar model of institutional psychosocial care exists and provides a balance between accessibility and accountability. The AI-based first contact is the first pillar, which is an emotional expressive conversational agent with no barriers to encourage first disclosure and encourage emotional expression via the use of empathetic response models (Inkster et al., 2018; AlMakinah et al., 2024). The second pillar, which is human-led escalation, makes sure that the complex or high-risk cases are referred efficiently to trained counselors who are in a position to provide the contextual knowledge, ethical control, and specialized intervention to them (Coghlan et al., 2023; Pasupuleti, 2025). The third pillar is committed to data-based prevention, which uses aggregated and anonymized patterns of interactions to detect early warning signs and make decisions to inform proactive institutional policy. These pillars, combined, would form a feedback loop where the AI ensures immediacy and anonymity whereas human experts would offer empathy and moral judgment as institutional wellbeing interventions would evolve and be enhanced as data-driven insights would accumulate.

Despite the fact that various studies are examining the concept of AI-based emotional support systems (Inkster et al., 2018; Fitzpatrick et al., 2017), there is a paucity of studies examining the hierarchical structures integrating the use of automated empathy with the presence of human supervising and the establishment of institutional control. There is scant literature available that looks at the interaction of these layers to ensure user trust, privacy, and psychological safety in the organization (Coghlan et al., 2023; Weidinger et al., 2021).

Based on these driving forces, the current study aims to explore how a hierarchical AI agent can be developed to function effectively as the cornerstone of a multi-pillar institutional support framework for psychosocial wellbeing. To explore this objective, we begin by examining existing literature on conceptual frameworks, AI-facilitated emotional support, ethics, and the trust dynamics of users, and proceed to describe the theoretical system architecture and design principles of the proposed SocialWellbeing framework before conducting an empirical analysis on crucial behavioral trends, namely the paradox of proportionality in AI-facilitated disclosures. The paper concludes with a discussion of its implications and limitations.

## 2 Theoretical framing and related work

### 2.1 Conceptual framework

Recent research in the field of artificial intelligence and digital mental health points to the fact that AI systems used in psychosocial settings should be closely regulated, ethically oriented, and integrated into the human support system. Instead of acting as independent therapeutic elements, AI technologies are also conceptualized as assistive technologies working under the framework of limited and controlled contexts.

Research on AI alignment claims that systems working on socially sensitive tasks ought to incorporate clear human values, institutional protections, and normative limitations in their design (Gabriel, 2020). When applied to mental health, ethical considerations place more emphasis on proportionality, transparency and the need to restrict autonomous decision-making in high-stakes situations (Coghlan et al., 2023; Wies et al., 2021). Empirical research has shown that conversational agents can supply available and stigma-reducing assistance to persons with anxiety or depression (Fitzpatrick et al., 2017), however, such systems can best be effective when they have their role delimited and have their functions governed by governance.

Similar studies reinforce the need to have human supervision in digital mental health ecosystems. The models of hybrid human-AI collaboration place artificial agents as assistive tools, not substitutes of professional judgment (AlMakinah et al., 2024; Wang et al., 2021). Human-in-the-loop models highlight the areas of accountability, escalation routes, and organized response when sensitive or high-risk scenarios occur (Pasupuleti, 2025). The need is especially acute in the context of adolescent mental health and bullying, where studies have repeatedly reported close relations between victimization and poor psychological outcomes (Man et al., 2022; Han, 2025). Research also suggests that there can be social and emotional repercussions to reporting experience (O'Brien et al., 2018), which confirms the significance of organized, empathetic human follow-up.

In addition to the support on the individual level, a new body of research poses the significance of institutional learning in digital wellbeing systems. The design-oriented research states that interaction-level data needs to be converted into system-level insights through digital wellbeing technologies. Research studies of the overall influences of AI in the educational context also focus on the tracking of aggregate psychosocial trends to drive preventive and policy action. Governance studies also emphasize auditability and feedback systems as two critical elements of responsible AI implementation in psychosocial care.

Combined, the literature narrows to three core criteria of responsible AI-enabled psychosocial support including value-constrained AI interaction, structured human supervision of severe or complex cases, and institutional learning through aggregated analytics. All these theoretical strands serve to inform the system architecture suggested in the next section.

### 2.2 Human-computer interaction for sensitive contexts

It is crucial to fully understand vulnerable users' emotional needs, their cultural differences, and the potential risks that could affect their safety and wellbeing before designing technology for them. In Human-computer interaction (HCI), three key principles must be followed: building trust, supporting user agency, and designing with empathy.

These are sensitive contexts, such as in mental health, where building trust among victims is a first and most important step to encourage the victims to share their experiences. Consequently, most victims would not be willing to report problems candidly due to fear of stigma or misuse of data. The previous studies show that users tend to trust computer systems when they are transparent about themselves for both capabilities and limitations (Pasupuleti, 2025), while systems seen as unpredictable or robotic rapidly reduce that earned trust (Naik et al., 2025; Bickmore et al., 2010).

The other core property is user agency. That is, systems should afford vulnerable users meaningful choices about how they use the system, what information they share, and whether to escalate issues to human professionals. Researchers emphasize that HCI should provide control to the users, rather than telling the users what to do (Al-Mansoori et al., 2023). Any system that conceals its options or defaults to automated responses may lead to feelings of powerlessness in users who already find decision-making challenging, especially in their daily lives for keeping things calm.

Last but not least, an empathetic design is truly about valuing users' emotions and understanding the struggles they go through-not just polite words (AlMakinah et al., 2024). Research into mental health chatbots shows users prefer kindness and sincerity over forced cheer and scripted responses, which often leave them feeling disappointed (Al-Mansoori et al., 2023). Jokes or casual remarks that fall outside of the situation will appear uninterested and dismissive instead of comforting. Generally speaking, it follows from everything the evidence underlines that technology created for sensitive situations has to be transparent, give people a sense of control, and show real care if it is to be of ultimate assistance (AlMakinah et al., 2024).

These design principles reflect the value alignment requirements outlined by Gabriel (2020) and sit within the graduated support structure of the stepped-care model (Andersson et al., 2019).

### 2.3 Ethical and practical considerations in AI-based psychosocial support

To assist the victims of bullying, harassment, and emotional distress in the educational and institutional setting, an emerging field of artificial intelligence (AI) implementation was designed termed psychosocial care. These AI applications, particularly conversational agents, have a number of benefits: they are anonymous, 24/7, and non-judgmental, meaning that they can help decrease the social barriers and stigma that are usually linked with seeking human assistance (Bickmore et al., 2010; Fulmer et al., 2023). Researchers have demonstrated that chatbots run using AI can be used to assist individuals in explaining their emotions, detecting distress patterns, and performing guided coping, and result in significant but relatively small changes in emotional wellbeing (Fitzpatrick et al., 2017).

But the applications of AI in such sensitive settings are fraught with a lot of ethical and technical issues. One of the most significant concerns is the value alignment; the AI should be able to operate in line with the moral and cultural values of the institutions and communities it operates (Gabriel, 2020). In the absence of such alignment, automated advice will tend to undermine systemic problems or strengthen inequalities (Coghlan et al., 2023; Burr et al., 2020). On the same note, algorithmic bias is dangerous, with AI models, which are trained on limited or western-centric data, being insensitive to linguistic or cultural manifestations of distress, making them less inclusive and less accurate (Thakkar et al., 2021; Weidinger et al., 2021).

Equally urgent are the data protection and privacy. The psychosocial communications usually deal with the highly personal revelations; thus, the insufficient anonymity or data abuse may lead to actual harm (Weidinger et al., 2021). In this regard, institutions need to maintain open data management and tight control. Ethical frameworks advise that AI must not be used in isolation but those systems with humans-in-loop, where professionals are in charge of making decisions, escalation, and care delivery (Wang et al., 2021; Naik et al., 2025; Pasupuleti, 2025). This maintains human judgment even on urgent cases, and is accountable.

In a practical perspective, a research also finds problems with depth of emotion and contextual perception. Chatbots, as reassuring and empathetic, usually generate automated responses that may appear cold or do not see crisis-level distress (Pichowicz et al., 2025). The use of such systems too much may result in false belief or emotional dependency which may discourage a victim to seek human assistance where needed (Sabour et al., 2023).

Thus, the new consensus in the digital mental health literature supports a multi-layered model with the first, low-barrier access point being AI agents, and the second, third levels, with human counselors and institutional infrastructure dealing with escalation and follow-up (Andersson et al., 2019; Wang et al., 2021). This makes sure that the effectiveness and availability of AI can be balanced with the emissiveness, situational thinking, and fairness of human specialists. Such frameworks, however, can be used responsibly to increase institutional capacity in the psychosocial provision without affecting the safety, trust, or quality of care.

This consensus supports the hybrid human–AI model (Wang et al., 2021), where AI serves as a structured collaborator and escalation remains proportionate to case severity (Andersson et al., 2019).

## 3 Methodology: the SocialWellbeing system as a research instrument

This section presents the methodological basis of the SocialWellbeing System, which was the central tool used for this research. The system is aimed at intervening on psychosocial harm in institutional settings with a three-pillar architecture that combines artificial intelligence, human monitoring, and data- driven prevention. All aspects of the system were preceded by methodological choices balancing technical effectiveness with ethical accountability.

### 3.1 The three-pillar research architecture

**Figure 1:**
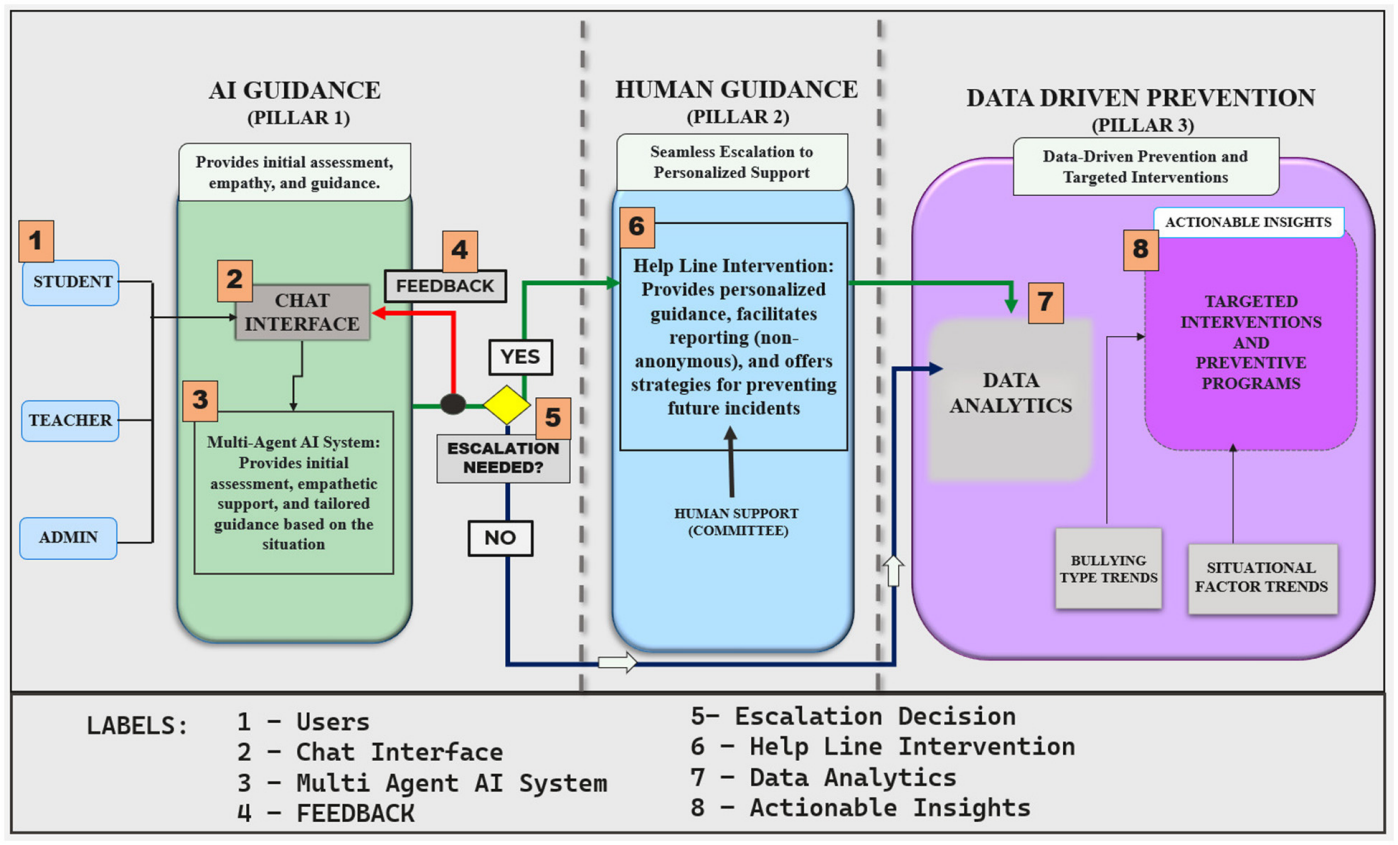
AI social wellbeing.

Figure 1 illustrates the overall workflow of the SocialWellbeing system. The numbered components (1–8) enclosed in curly brackets{} represent the sequential operational stages as follows:

- **{1} – Users:** Institutional stakeholders such as students, faculty, and staff who interact with the system.
- **{2} – Chat interface:** The conversational interface enabling real-time interaction.
- **{3} – Multi-agent AI system:** The coordinated AI agents responsible for response generation and analysis.
- **{4} – Feedback:** User feedback loop for system refinement and adaptive learning.
- **{5} – Escalation decision:** User's decision to escalate the case for human support.
- **{6} – Help line intervention:** Human expert support triggered for all the cases escalated in order of empathy score.
- **{7} – Data analytics:** Analysis of user interaction data for institutional monitoring.
- **{8} – Actionable insights:** Policy, rules and preventive strategies uploaded by institutions.

Figure 1 depicts the high-level architecture of the SocialWellbeing system and illustrates how institutional stakeholders, including students, faculty, non-teaching staff, and administrators{1}, interact with the platform. All user groups access the system through a conversational chat interface{2}, which serves as the primary point of engagement. Administrators additionally define institutional policies, rules, and escalation thresholds, embedding governance and ethical oversight directly into the system's operation. User interactions are processed across the system's three pillars, providing an integrated view of assessment, support, and prevention.

The first pillar, **AI guidance**, is the system's first point of support. Users get prompt evaluation, considerate answers, and personalized advice via the chat interface. For instance, a faculty member reporting harassment is linked to the appropriate support services, and a student experiencing academic stress receives guidance tailored to their need. This layer is based on a multi-agent AI system{3} that provides the opportunity to evaluate user input and identify the nature of an issue reported, emotional state, and generate empathetic and context-sensitive responses.

The process of escalation to the second pillar (path of the green arrow) is controlled by a multi-factorial mechanism and not an one-factor trigger by the user. In the case of persistent vulnerability, the AI kindly notifies the user about the presence of human support and introduces an escalation{5} option. Once selected, the user is taken through a short series of close-ended questions, meant to measure emotional distress, coping ability, and readiness to get help. The responses are calculated to give a score on emotional risk, which enables the system to distinguish between real psychosocial vulnerability and temporary frustration. Notably, it will escalate only with final escalation confirmation (yes/no). If the user wants to escalate (path of the green arrow), they are asked to fill out the escalation form, which involves the contact details like their email address, phone number, and department which is not mandatory, this information with the emotional score is sent to Pillar 2. In case a user does not want to escalate (path of the blue arrow), the communication does not leave the AI Guidance layer and is guaranteed to deliver autonomy and ethical transparency.

The system also includes a structured feedback loop (shown using the red arrow) that is placed immediately after the escalation confirmation (Yes/No) step {4} where the user is asked to provide feedback on whether the AI answer was helpful. This feedback is not used to automatically escalate the issue but is instead used to evaluate the effectiveness of the interaction, improve prompting approaches, adjust the empathetic tone, and re-evaluate the user's emotional state or level of urgency. The user maintains full control to either confirm escalation or cancel.

The second pillar, **Human guidance**, deals with the cases involving personal and real-world intervention. After the approval of the escalation, a brief summary of the incident and the anonymous emotional risk score is sent to trained human supervisors or advisors. The cases with low emotional scores are given high priority so that those who are more vulnerable are attended to at a quicker rate without wasting human resources. This human-in-the-loop design{6} guarantees that users receive individualized attention and avoids an excessive dependence on automated responses in dangerous or urgent circumstances.

The third pillar, **Data-driven prevention**, brings together data analytics {7} with actionable insights {8}. Aggregated trends, such as bullying patterns and situational risk factors, are systematically analyzed to guide institutional policy updates, rule refinements, and response-governance adjustments. The core functional pipeline of systems and structural processes will not change. It is important to note that the system has a direct link between Pillar 1 (AI Guidance) and Pillar 3 (Data-Driven Prevention) (shown using blue arrow). This link is triggered whenever the user decides not to escalate or when escalation is indicated but not confirmed. In such a scenario, the interaction will not go to human review; instead, anonymized conversational metadata and user feedback are submitted to Pillar 3 for analysis.

In Pillar 3, non-escalated interactions are analyzed to determine patterns, stress signals, or dissatisfaction with AI assistance. The data obtained is then used to improve response strategies, escalation thresholds, and interaction quality under supervised analysis. This ensures that even when there is no escalation, every interaction is used to optimize the system continuously and generate preventive insights.

By incorporating organized escalation, feedback-driven AI adaptation, and an element of continuous institutional learning, the SocialWellbeing system works as a closed-loop socio-technical system. Personal interactions lead to preventive intelligence, institutional understanding enhances AI directions and management, and specific interventions decrease future psychosocial threats, which makes the environment safer and more responsive to all stakeholders. The system has an elementary user feedback system to know whether the AI response was useful and whether there was an escalation on the basis of the inadequate AI support or the user needed extra assistance. According to this feedback of the interaction level, the AI can rephrase the guidance, change its empathetic tone, or ask a question when the distress continues. This can assist in making sure that escalation is used to indicate the real needs of the user in relation to support not just the inability to provide the response.

#### 3.1.1 Overview of the SocialWellbeing system workflow

**Figure 2:**
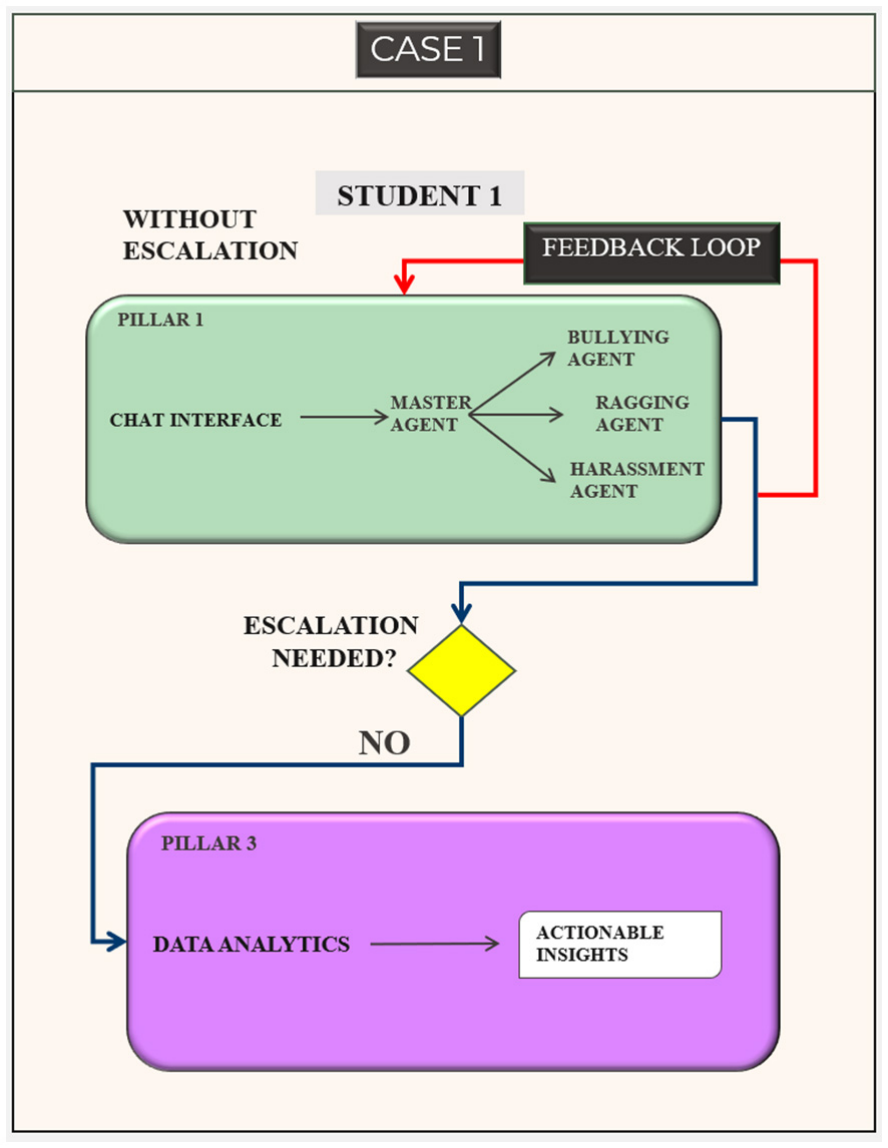
Student–system interaction without escalation.

**Figure 3:**
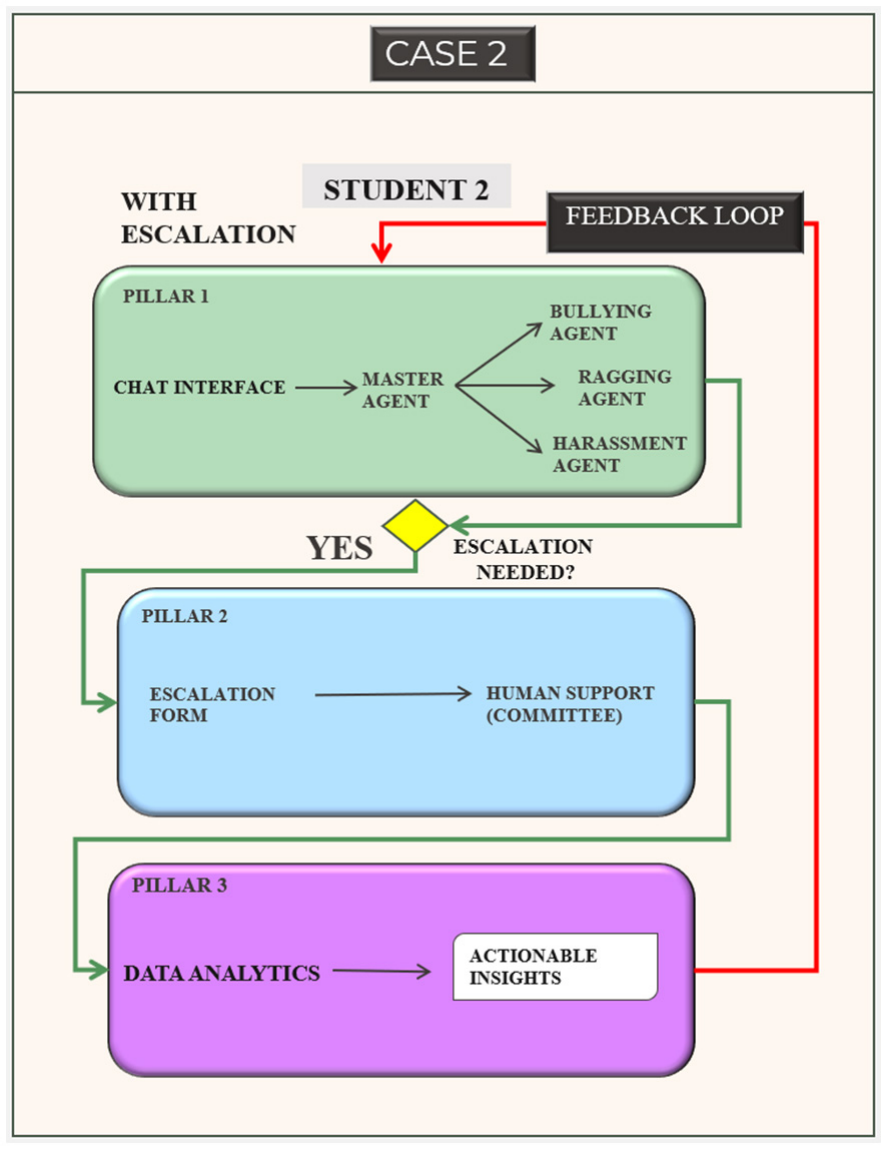
Student–system interaction with user-consented escalation.

The general workflow of the SocialWellbeing system is illustrated in Figures 2, 3 with the interactions between students and AI. Communication begins with the chat interface, which is the entry point of the system by a student. An AI agent organizes the message with a master agent and then the message is categorized into a list of bullying, harassment, ragging, or general wellbeing by the agent. This process is assisted by a set of specialized sub-agents which extract and use contextual and emotional indicators to inform risk assessment.

In the first case (Figure 2), the message of STUDENT 1 is evaluated as a low-risk message and the student decides not to escalate. With the help of the AI Guidance pillar, the students receives a understanding and contextual guidance, as the case is not escalated the communication stops at Pillar 1. This anonymized conversational information is then directly sent to the Data-Driven Prevention pillar (Pillar 3), where they are analyzed to create actionable insights that can be used to support institutional awareness and preventive planning.

In the second case (Figure 3), the message is classified as high risk and STUDENT 2 specifically chooses to escalate. The system offers the organized escalation option in the chat box, and upon the approval of the student, the feature directs the student to fill out the escalation form, which involves the contact details like email address, phone number, and department and other details. This triggers the Human Guidance pillar (Pillar 2) where trained human supervisors are able to go through the case, contact a student and offer the suitable real world support. This will make sure that the process of escalation is user-driven and managed in a human way so that the necessary intervention can be effected in case of any further support.

In both cases, user feedback is gathered to help in the continued improvement of the system. This feedback comprises the helpfulness of the AI responses and the presence of escalation because of the lack of AI support or the necessity of the user to have additional support. These inputs are reconsidered to understand how to make timely design, enhance transparency of the response and enhance a better calibration of the escalation criteria. Such a human and controlled feedback mechanism helps in responsible system development and user autonomy coupled with proper ethical control.

### 3.2 Technical implementation and responsible AI framework

**Figure 4:**
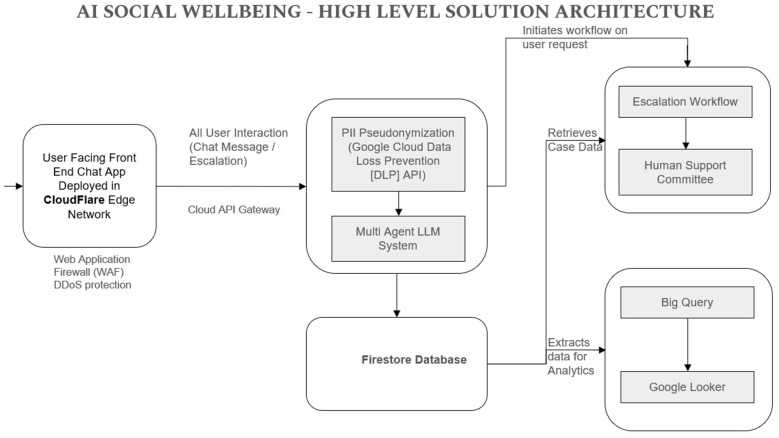
AI social wellbeing—high level solution architecture.

**Figure 5:**
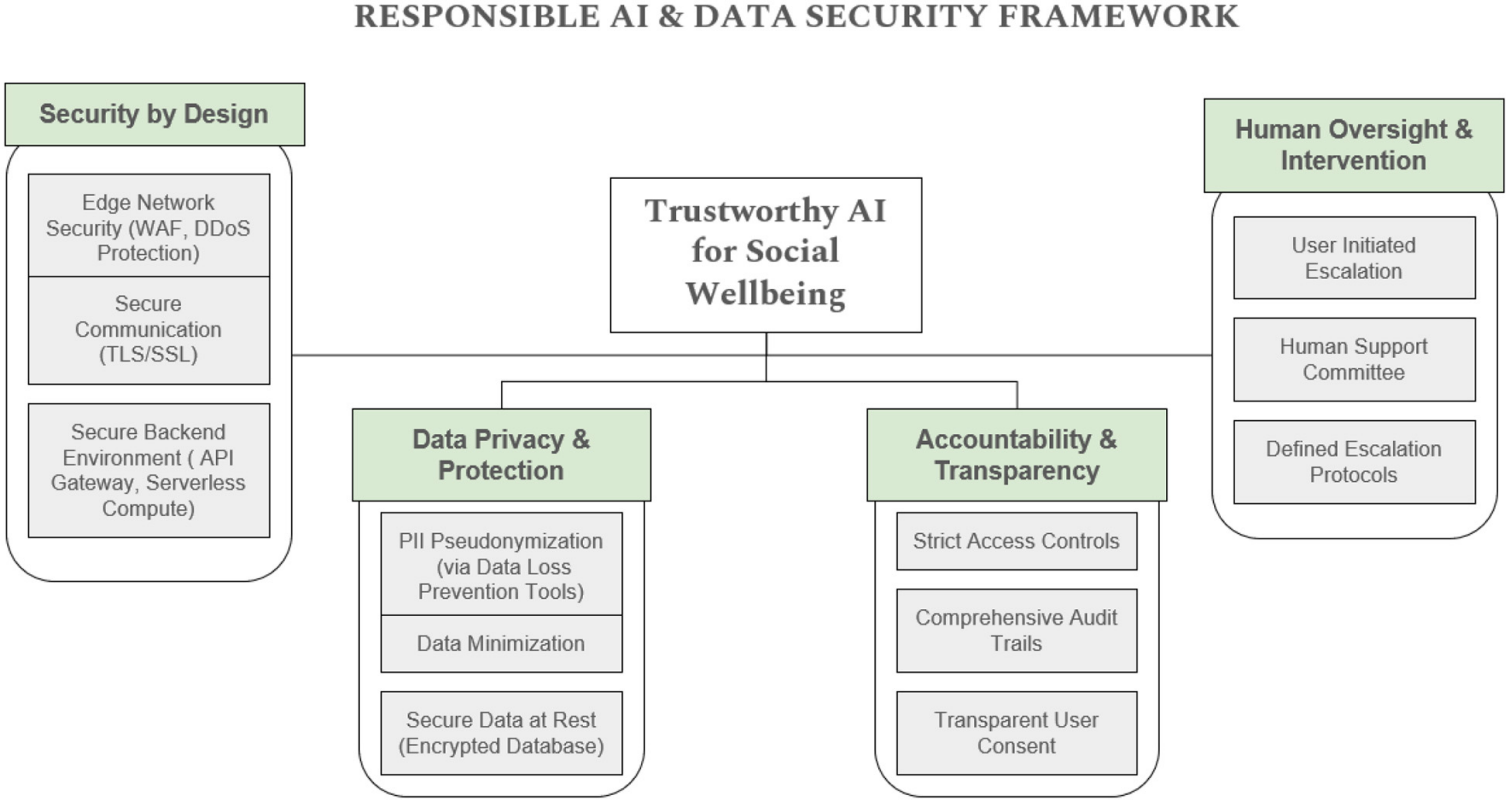
Responsible AI and data security framework.

The Social Wellbeing System is integrating the advanced cloud architecture together with multi-agent AI designs to provide security and enhance it's responsiveness. Figure 4 illustrates the complete flow of data and it's processes of the system, while Figure 5 highlights the Responsible AI and Data Security Framework showing the system's design.

#### 3.2.1 System overview

The AI Social Wellbeing high-level solution architecture is depicted in Figure 4. It defines user interaction with the system, flow of data between components, as well as security checking at each stage. When one of the users initiates a dialogue, it happens in a chat application, which operates on the Cloudflare edge network. This implementation ensures the use of a Web Application Firewall (WAF) and mitigation of the DDoS attack of malicious traffic. Any chat message or escalation request passes through a cloud API Gateway. The gateway is a secure transmission point that forwards traffic in between the user interface and the backend. The system passes the message via Google Cloud Data Loss Prevention (DLP) APIs to pseudonymise personal data before the Large Language Model (LLM) processes it in the system. This conceals recognizable data at an earlier stage. Once cleaned, the message is passed through the Multi- agency LLM system which intelligently processes, risk-analyses and classifies the message. The anonymised results are also stored in Firestore (Firebase Database) only after this stage has been completed. Within the Multi- Agent LLM system, various agents in the system appraise the inputs made by users in areas like wellbeing, harassment, ragging, or bullying. This is because the AI provides a guided response or raises an issue to be reviewed by a human depending on the risk and the circumstances. Figure 4 AI Social Wellbeing -Tablet Level Solution Architecture.

Firestore is the secure storage of all structured and anonymised data. At this point, the data is either processed down one of two paths:

1. Escalation workflow: the appropriate information about the case is supplied to the Human Support Committee that addresses issues related to sensitive cases in real time.
2. Analytics and reporting: data is migrated to the BigBank Data Analysis platform to be analyzed on a huge scale and presented in Google Looker dashboards and provide the institution with insights regarding stress levels, patterns of harassment, and preventive measures.

This technical architecture is a compromise between speed, data integrity and ethics control that rids them with stable support keeping user data confidential.

#### 3.2.2 Responsible AI and data security framework

Figure 5 responsible AI and data security framework includes the architecture of the SocialWellbeing system adjusted to the principles of ethics and privacy protection. It also makes sure that all of the components have fairness, transparency and human accountability.

This framework is structured around four core dimensions:

(1) Security by design: It has a layered protection; WAF and DDoS protection on the edge, encrypted TLS/SSL channels, and serverless API gateways. These controls ensure that the systems will be of high integrity and can achieve high availability.

(2) Data privacy and protection: The automated protection of PII pseudonymization (through DLP tools), stringent data minimization, and encryption is carried out on the user data at rest and in transit. Only necessary information is gathered and anonymised then analyzed and strengthens trust and privacy regulations.

(3) Accountability and transparency: Audit trails and tight access controls are used to track all backend interactions. Clear consent forms are also built into the chat interface, which gives users an opportunity to learn how their information is utilized and consent to it.

(4) Human oversight and intervention: Human Support Committee looks at cases marked by the AI or cases submitted by the user. Well-defined escalation policies will guarantee that all high-risk or ethically sensitive situations will be handled by a human being, and will keep empathy and control in decision-making.

When combined, these four principles will guarantee that the SocialWellbeing System will serve not only as a technical instrument but as a *concept of responsible AI implementation*. The design will instill a sense of security, privacy and fairness by placing it at its core, thus enabling the user trust and institutional responsibility.

**Figure 6:**
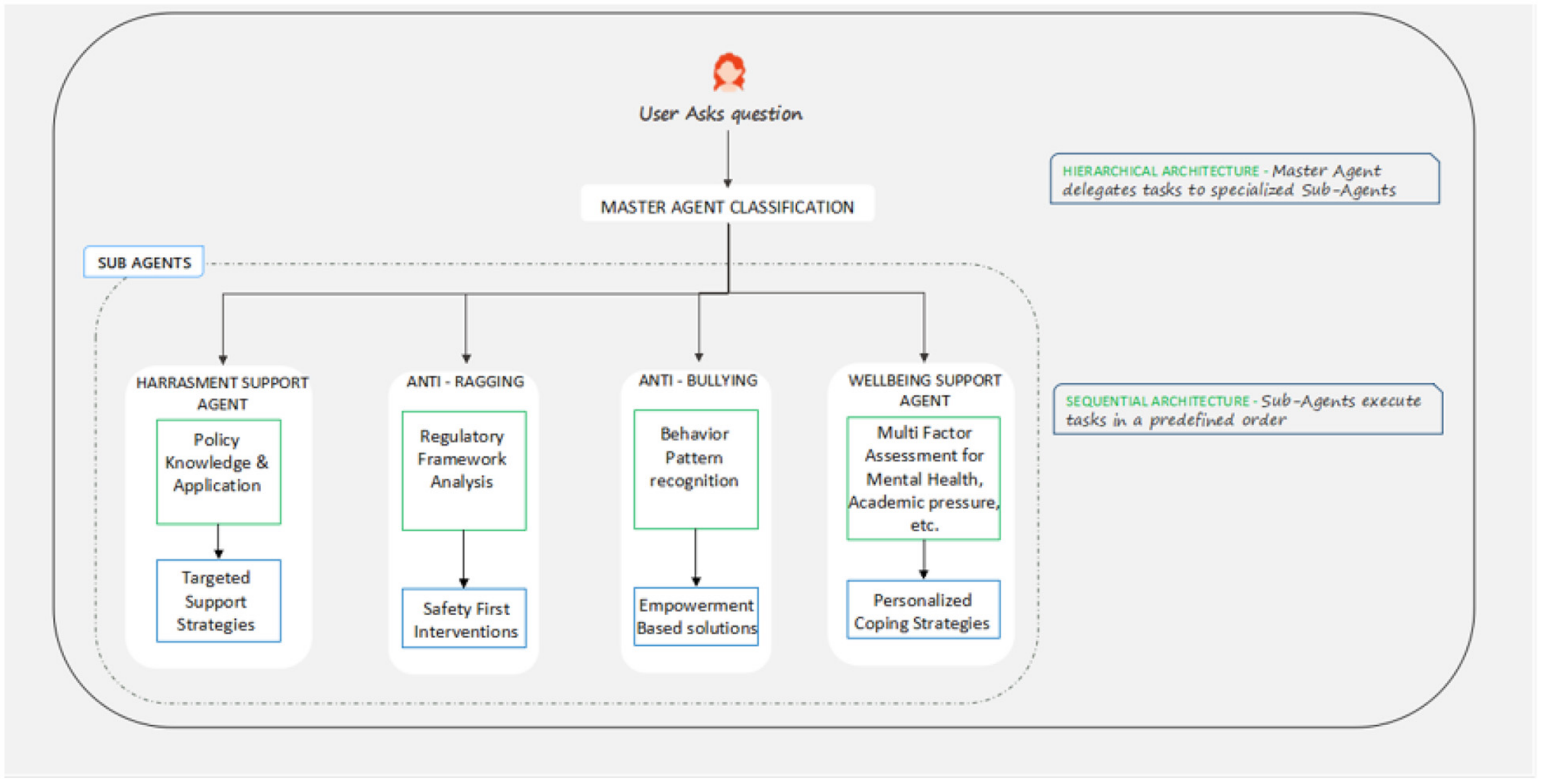
Master agent classification.

Pillar 1: multi- agent AI guidance system. The first pillar of the system forms the basis of the intelligent response of the SocialWellbeing System. This is the layer, as in Figure 6, which is known as the MasterSub- Agent hierarchical architecture and enables, specialization, controlled growth and interaction management, which is adaptive.

#### 3.2.3 Hierarchy architecture overview

When a message is posted by a user, it passes through the system first through the Master Agent that undertakes a review of the message to identify the category of wellbeing of the user, i.e. general wellbeing, harassment, bullying, or ragging. Instead of using traditional methods of natural language processing, such as intent detection or semantic parsing, this model makes use of large language reasoning with Chain-of-Thought prompting, an internal knowledge base of institutional policies, and the A.G.E. framework. This interaction enables the system to think through user input just like a trained human counselor would intuitively and intuitively- through interpretation of context, emotion and inferred need resulting from input instead of on simple keywords.

Every conversation session has its own unique Session ID that is the continuity of the conversation and makes it traceable. The Master Agent is constantly appraising its confidence in the classification, understanding it better as the conversation continues. Once its confidence exceeds a set value (usually 0.8 or greater) the conversation is automatically handed over to a dedicated Sub-Agent that is specific to the particular type of concern.

Where this is less than the confidence, the supportive Buddy Agent temporarily assumes control. This agent will interact with the user into clarifying questions and brief interactions, try to elicit more context in order that the classification can arrive at a consistent and correct decision. The conversation can be refined and sent back to the Master Agent to get reaffirmed and then has to be routed to the appropriate Sub-Agent.

Such hierarchical arrangement provides collaboration and coordination between agents- all of them have a distinct, premeditated role. The hierarchy encourages adaptive interaction, consistency in support quality, and responsible information handling. More importantly, it keeps the system aligned with institutional values: empathetic engagement, informed reasoning, and human oversight at every significant decision point.

#### 3.2.4 Sub-agent specializations

In order to solve various psychosocial issues, the system combines five specialized Sub-Agents, each of which is optimized in one direction and has certain decision logic:

1. Harassment support agent: Deals with interpersonal and institutional harassment in three sub-modules:

- *Knowledge and policy application:* Routes reports to institutional or legality.
- *Support strategies: targeted:* Offers step-by-step instructions to student at-risk, active, and talented students.
- *Safety-first interventions:* Switches on safety or protective measures where necessary.

2. Anti-ragging agent: It aims at preventing and addressing cases of ragging.

- *Regulatory framework analysis:* Removes non-compliance with anti-ragging norms in institutions.
- *Sustained action:* Provisions short-term safe-exit or escalatation opportunities that are safety-oriented.

3. Anti-bullying agent: Identifies bullying habit in either academic or social settings.

- *Behavior pattern recognition:* Recognizes isolating or serial aggression.
- *Empowerment-based solutions:* Proposes peer-support systems and proactive response structures.

4. Wellbeing support agent: Resolves emotional, academic, and mental health issues.

- *Multi-factor assessment:* Measures interpersonal, environmental, and task stressors.
- *Individualized coping plans:* Prescription of schedules, mindfulness, and institutional counseling channels.

Every Sub-Agent works independently but with the ability to delegate cases to other parties in the future. As an example, a bullying case, which escalates to be a harassment case, is automatically transferred to the Harassment Agent, so that there is a continuity in the user support.

#### 3.2.5 Prompting framework and refinement

The Master and Sub-Agents were also trained and tested in two development stages:

- Phase 1: Few-shot prompting. The first prompts were geared toward rule-based classification with few-shot examples which entailed each Sub-Agent being given 12 case-specific scenarios to reinforce logical reasoning.
- Phase 2: Chain-of-thought prompting. Based on Phase 1, step-wise reasoning was added to prompts, which allowed the systematic decision trees and explanatory answers. This brought great increase in interpretability, consistency, and empathy of Sub-Agent behavior.

These prompting strategies were all based on the Gemini Flash Lite 2.5 model but the final one arrived after the comparative consideration of Gemini Flash 2.0, 2.5, Flash Lite 2.0, and Pro 2.5. Structured metadata was applied to each model output, such as: Agent Type, User input, Bot Type Response, Confidence Score, Escalation Flag, Response Time, Timestamp, and Expected Outcome. This schema enabled analysis of performance systematically and gradually improved it.

#### 3.2.6 Sequential or hierarchical coordination

In the agent ecosystem, the following two coordination mechanisms are used:

1. Hierarchical delegation: The Master Agent sends messages to Sub-Agents depending on classification Knowledge and policy reasoning.
2. Sequential processing: Sub-Agents are guided by established procedural processes analysis, guidance and escalation processes, making sure that all interactions are handled according to institutional workflow.

This hybrid design of coordination is more scalable. Introducing New Sub-Agents can be done in a modular manner without having to retrain the entire architecture to facilitate flexibility in meeting the changing needs of the institution.

#### 3.2.7 Fairness, interpretability, and scalability

The Pillar 1 design balances between three key objectives:

- Scalability: Allows the dynamic deployment of new agents to emerging categories of issues.
- Fairness: The Master Agent is a confidence-based router that eliminates bias and results in fair treatment of cases.
- Interpretability: Chain-of-thought reasoning develops the human reviewability of AI is achieved through the provision of supervisors to track the decision-making process of AI.

In general, Pillar 1 brings the idea of AI-based psychosocial guidance into practice. It transforms conversational data to structured decision making procedures that assist in real time coordinated responses in psychosocial contexts.

### 3.3 The two-phase evaluation protocol

The SocialWellbeing System was tested in two successive stages that were aimed at accomplishing and enhancing the technical consistency of the system, as well as the social-emotional performance. The systematic review explored the model precision, consistency of empathy, and latency on a representative sample of psychosocial cases.

#### 3.3.1 Ethical oversight

This evaluation used anonymized, fictional scenarios created by students. No real personal information or actual case records were involved at any stage. Two study authors- one industry expert and one super vising professor-independently reviewed and labeled the scenarios. Any differences in their assessments were discussed and resolved together.

#### 3.3.2 Scenario creation and labeling

Sixty anonymized campus scenarios were created by student contributors as fictional, depersonalized vignettes portraying situations involving bullying, harassment, ragging, peer conflict, and wellbeing concerns. No real personal data or live case records were used at any point.

A five-member panel—consisting of the three student co-authors, an industry expert, and the super vising research professor—independently reviewed and labeled each scenario. The initial labels were assigned individually. Final label decisions were agreed upon through a consensus meeting between the industry expert and the supervising professor. Individual rater notes were not retained.

The anonymized scenarios and the final consensus labels can be provided to the Editor upon request.

#### 3.3.3 Phase 1: baseline evaluation

The initial step has created a reference point of the basic behavior of the system. The experimental protocol was a replication of experiments of various Gemini-family language models (Gemini -Pro, Gemini -2.5-Flash, Gemini -2.5-Flash-Lite, Gemini -2.0-Flash, and Gemini -2.0-Flash-Lite) on anonymised, pre-curated set of psychosocial interaction examples. All prompts represented real-world simulation dialogue situations like harassment complaints, academic stress, interpersonal conflict, and ragging situations. In the case of each test case, the master agent of the system conducted three vital functions: (1) initial classifications of intents, (2) response generation with empathy weight, and (3) session control. To ensure consistency, all trials were carried out with the same session-ID-based pipeline.

For every test instance, the system's master agent performed three critical tasks: (1) initial intent classification, (2) empathy-weighted response generation, and (3) session management. To ensure consistency, the same session ID-based pipeline was applied across all trials.

Evaluation metrics:

- Classification accuracy: Measured as the ratio of correctly identified issue categories to total cases processed.
- Response quality: was tested on a five-point scale of rubric that included the clarity of the response, the applicability of the response to the context, empathy, the accuracy of the guidance and the linguistic consistency.
- Latency: The average time (in seconds) that it took to generate the first token upon reception of the message was recorded.

Findings: Phase 1 showed that the system worked effectively in the normal cases having explicit intent cues. Nevertheless, there had been lower performance with ambiguous cases or with multi-layered cases. The compassion on the reaction of different model types was varying with Gemini-Pro having the highest percentage of model contextual knowledge along with the latency. Gemini1.5-Flash produced faster response with lesser quality. Such revelations highlighted the strength of initiating restrictions and contingent back-up strategies.

#### 3.3.4 Phase 2: intervention and re-evaluation

After the initial analysis was completed; Phase 2 introduced the systematic technical interventions to improve accuracy and dependability. The strategy was sensitive to structured prompt engineering, model optimization and control that had confidence-based responses.

The following enhancements were implemented:

1. Strict rule-based prompting: Every agent was provided with a deterministic input format, which specified the limits of domains (harassment, bullying, ragging, and academic wellbeing). This minimized inter-domain mix-up in classification.
2. A.G.E. framework integration: the answers were resolved through three steps logic: Acknowledge (empathy and validation), Guide (contextual advice or support), and Escalate (referral to human assistance in case the confidence threshold is less than 0.8).
3. Confidence-based fallbacks: master agent used a confidence score C ∈ [0, 1] based on each classification. When the C was below 0.8, user input was sent to a Buddy Agent that reduced query intent by a series of short iterative questioning and routed.

Post-intervention evaluation: The model pipeline which was improved was re-assessed with the same test conditions. Results indicated for Gemini 2.5 Flash models:

- Improved classification accuracy, with phase 2 models achieving 88%–89% compared to Phase 1 baseline of 84%–86%.
- Enhanced empathy scores, with Phase 2 models rated 4.4–4.8 compared to Phase 1 scores of 4.2–4.3 on a 5 point scale as rated by human evaluators.
- Increased response times of 20–25 seconds in phase 2 due to more sophisticated chain-of-thought processing, compared to Phase 1 range of 7–20 seconds for Gemini 2.5 Flash models.

Moreover, the promptness inclusion of the A.G.E. framework to a great extent increased the clarity of the structure of generated responses and provided users with confidence when a response is needed within extended sessions. The confidence based fallback mechanism ensured that there was no misclassification cascades, and the escalation handovers to human support were smoother.

Summary: The two-stage assessment plan confirmed the evolutionary adaptability of the system, starting as a simple prototype of the basic functional model and moving on to an effective and situational-aware AI guidance support system that is morally insulated. These process optimizations prove that the SocialWellbeing System is both technically scalable and socially responsive and can be accurately implemented in an institutional setting.

## 4 Findings

This section reflects the findings of the overall evaluation of the Social-Wellbeing System, performed in the context of its two-stage evolution and 3-pillar system of operation. The discussion has been done on the Master Agent (the main conversational AI to interpret and classify user inputs) and the Sub-Agent that assists in contextual reasoning and advice. In addition to the performance of conversation, the assessment checked the mechanisms of Pillar 2 (Human Expert Escalation) and Pillar 3 (Data-Driven Prevention Analytics) to determine how the system works as a supportive network of socio-technical performance.

Sixty curated test scenarios were utilized and they modeled real world conditions of campus wellbeing, including the following scenarios: bullying, harassment, discrimination, peer conflict, and mental-health distress. Quantitative and qualitative metrics were used to evaluate each scenario in terms of accuracy, latency, classification reliability, contextual alignment, and perceived helpfulness with controlled model parameters.

The assessment was designed as follows and was divided into two stages based on models of the Gemini family:

- Phase 1 set the baseline with few-shot prompting architecture showing some major weaknesses in empathy calibration, guidance accuracy, and speed of response.
- Phase 2 brought a formal chain of thought prompting model, derived, though not limited, to the A.G.E. (Acknowledge-Guide-Escalate) logic, by adding rule-based reasoning and deterministic validation layers. This setup not only brought quantifiable gains in accuracy, coherence and latency, but also revealed a new difficulty: this system is over-sensitive to ambiguous inputs.

Moreover, the Pillar 2 results assessed the flow of escalation to human trained experts, completeness and confidentiality of information, and user experience during the process. Pillar 3 results were obtained based on a Looker Studio dashboard that visualizes the trends of the incidences, the patterns of escalation and indicators of early-intervention giving institutional understanding of the prevention measures.

Altogether, these results indicate that the system is iteratively refined during both stages, as well as that therapeutic balance is shifting, with respect to empathy, interpretability, and proportionality, in each of the three pillars.

The findings are presented in the following way:

- 4.1 Master Agent evaluation - Phases 1 and 2 Gemini models performance quantitative, accuracy, and response-time.
- 4.2 Sub-Agent evaluation - Determination of the quality of guidance and helpfulness, with the help of comparative graphs and marking of the evaluator.
- 4.3 Pillar 2 and Pillar 3 evaluation - Real-world escalation and prevention outcomes, which are demonstrated using communication logs and institutional dashboards.

### 4.1 Master agent evaluation

#### 4.1.1 Evaluation setup

The Master Agent is the most conversational AI which is in charge of understanding, categorizing, and assisting users in sensitive wellbeing situations. In this assessment, Gemini family models were employed because of free access to research, and such models could be reproduced and tested extensively. There was no use of openAI and Claude models since they could not be used in large-scale experimental evaluation due to licensing limitations and the cost of APIs.

The models that were tested during Phase 1 comprised of Gemini 2.5 Pro, Gemini 2.5 Flash, Gemini 2.5 Flash-lite, Gemini 2.0 Flash, and, Gemini 2.0 Flash-lite. The prompting technique presupposed a small number of examples, which are supposed to classify incidents, judge the tone of emotion, and give supportive advice. The parameters in the model were set to temperature = 0.7, top-p = 0.9, and top-k = 40.

The Phases 2 assessment concentrated on Gemini 2.5 Flash and Flash-lite, with a chain-of-thought structured prompt design, which incorporated deterministic validation rules based on A.G.E. logic (Acknowledge-Guide-Escalate). The parameters of the model did not change compared to Phase 1.

The test data included 60 natural learning real-life campus wellbeing scenarios, in five categories: Bullying, Harassment, Discrimination, Peer Conflicts, and Wellbeing Concerns. Each model is tested with the same 60 test scenarios to ensure a fair and consistent performance comparison. In this section, 3–4 illustrative examples are included in a table and the quantitative results give the performance in all scenarios.

#### 4.1.2 Phase 1 findings

Phase 1 proved that the initial Master Agent was able to express empathy under simple conditions, however, a number of important concerns have been noted. At this stage, the models were directed by few-shot prompting, when a limited set of illustrative examples were shown to assist the agent in the classification of incidents and assessment of emotional coloring and provide supportive guidance. Although this method enabled the system to capture the simple situational context, it could not deal with more complex or sophisticated situations.

Table 1 encapsulates average response time and accuracy of classification of each model.

**Table 1:** Comparison of Gemini models by response time and accuracy.

Model	Avg. response time	Accuracy
Gemini 2.5 Pro	50 seconds	88.31
Gemini 2.5 Flash	20 seconds	85.71
Gemini 2.5 Flash-lite	7 seconds	84.38
Gemini 2.0 Flash	7 seconds	73.6
Gemini 2.0 Flash-lite	6.5 seconds	74.75

**Figure 7:**
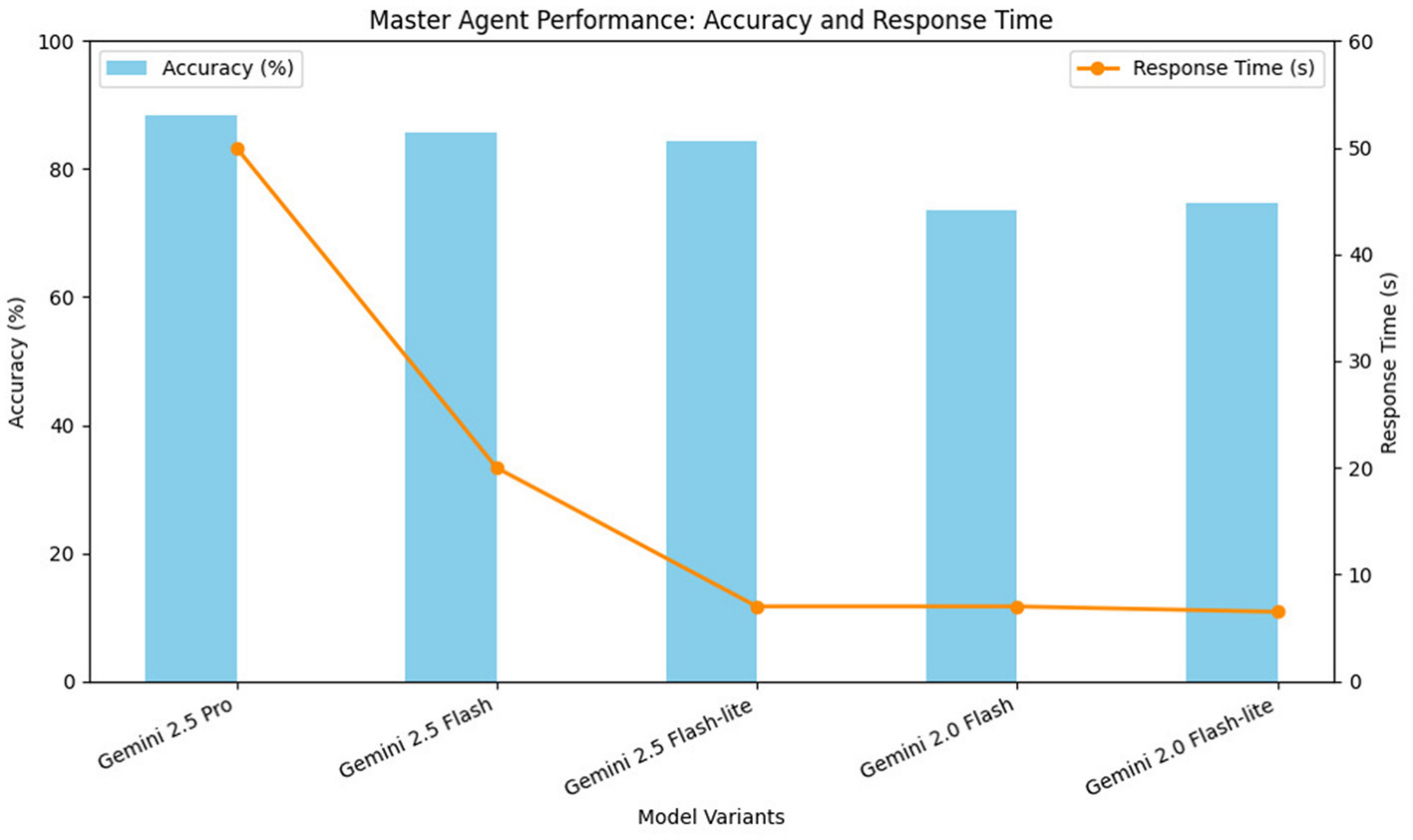
Phase 1 master agent performance: accuracy and response time.

As seen in Figure 7 that shows the graphical representation of Table 1, the most sophisticated classification performance was demonstrated by the Gemini 2.5 Pro that was able to detect intricate social and emotional signals properly. Nonetheless, its median turnaround time of average of 50 seconds made it inapplicable to real-time support environments, where users need instant information when they find themselves in distressing conditions. The Gemini 2.5 Flash and Flash-lite models, in contrast, offered a more reasonable trade-off between speed of response and accuracy, giving answers with a response of either 7–20 seconds, and the classification performance was also very good. The more rapid earlier versions of the Gemini 2.0 had significantly lower accuracy than the 2.5 Flash models were, so they were not as useful at classifying the nuances of wellbeing. One of the common problems in all the lower-accuracy models was that identity-based harassment, such as disability-related cases, would be classified as general bullying. This misidentification minimized the weight of the damage and did not allow having the right instructions to the users to institutional support like advisors or complaint committees.

One more urgent problem which was noted in Phase 1 was improper or unproductive guidance. When the agent showed empathy, sometimes the responses took previous events as urgent cases and advised users to contact crisis hotlines or campus safety. Although these responses are generally good, they would cause unnecessary stress and user trust would be negatively affected.

Lastly, the higher capacity models had slow response times, which further lowered usability. It will take almost a minute to get instructions during a potentially stressful situation, which only increases anxiety and destroys confidence in the system.

These results demonstrate, as a group, a trade-off in Phase 1 models: models with high capacity provided fine-grained insight but were slow, and those with high speed were responsive, but false-frequently and made unhelpful recommendations. Even though the few-shot prompting strategy was applicable in the simple cases, it was ineffective in handling complex or identity-based cases. The following challenges were the foundation of the Phase 2 improvements.

#### 4.1.3 Phase 2 findings (improved performance)

Phase 2 interventions were aimed at overcoming the main weaknesses noted during the Phase 1, i.e., misclassification of sensitive incidents, unhelpful guidance, and ineffective response time. To this end, the evaluation made use of rule-based chain-of-thought promoting and deterministic validation principles which enabled the Master Agent to have an organized logic in the interpretation, categorization, and reaction to intricate wellbeing situations. This method enabled the system to handle the input in a more holistic manner in a way that consideration was made to not only the emotional dimension of incidents but also the contextual dimension of incidents.

Table 2 represents the mean response time and the mean classification accuracy of the Phase 2 models.

**Table 2:** Comparison of Gemini 2.5 flash and flash-lite models by response time and accuracy.

Model	Avg. response time	Accuracy
Gemini 2.5 Flash	25 seconds	88.65
Gemini 2.5 Flash-lite	20 seconds	87.7

In comparison to Phase 1, misclassifications were more or less fixed. As an example, cases that might have been classified as general bullying like disability related harassment were now classed correctly as being harassment. This was enhanced so that the users could be given contextually relevant advice as well as referred to empowering institutional support measures, including counselors or grievance committees.

Also, the level of guidance improved with the introduction of the A.G.E. framework (Acknowledge, Guide, and Escalate). The structure of the Phase 2 responses was such that it initially recognized the distress experienced by the user and then proceeded to provide actionable steps to the user to handle the problem, scoring the need to report incidents or reach out to the corresponding support personnel and then lastly escalating the issues as considered necessary. The strategy generated not only empathetic but also practical responses to the situation that were both in line with the policies of the institutions and retained users trust.

Latency was also significantly decreased, with the Flash-lite version taking around 20 seconds to respond, and a reasonable trade-off between responsiveness and speed was found. This enhanced the system to be fit in real-time support where users get to feel they are heard and are supported without unnecessary delays.

Table 3 gives representative examples of Phase 1 failures and the corrective measures that have been made in Phase 2.

**Table 3:** Comparison of system classification across phases.

User query	Expected class	Phase 1 class	Phase 2 class	Outcome
“They keep calling me names and laughing at me during tutorials every week.”	Bullying	Bullying	bullying	No correction
“A student made derogatory comments about my disability and laughed about it.”	Harassment	Bullying	Harassment	Corrected
“My seniors asked me to sing in front of everyone, but I don't know if it was just for fun or something else.”	Ragging	Bullying	Ragging	Corrected
“I don't feel very included, but maybe I'm just overthinking.”	Wellbeing	Wellbeing	Bullying	New error

Phase 2 did lead to a major performance improvement but a new challenge, known as the Proportionality Paradox, arose. There was occasionally overinterpretation of the low risk or ambiguity user entries and this led to unnecessary increased responses. As an example, even weak self-doubt like remarks like, I do not feel very included, but I may be overthinking, were considered bullying which led to a stronger reaction, which was out of proportion to the situation. This effect underscores one of the design concerns of socially conscious AI: making AI more sensitive to grave events is bound to accidentally cause over-reactivity during low-risk situations.

On the whole, during Phase 2, structured prompting, deterministic validation, and the A.G.E. framework significantly enhanced the accuracy of the classification made by the Master Agent, the quality of guidance, and response time. Calibration is, however, required so as to achieve a balance between sensitivity and proportionality to ensure that the system is sensitive and contextually correct throughout the range of user inputs.

### 4.2 Sub-agent evaluation

The Sub-Agent was assessed in order to check the quality of direction and general usefulness of the guidance given when simulating a support conversation. While, the Master Agent was mainly aimed at correct classification and escalation, the Sub-Agent was created to maintain an empathetic conversation, action steps, and reassurance during user interactions.

#### 4.2.1 Evaluation setup

The same 60 simulated interaction scenarios were used to carry out the evaluation of the Sub-Agent for phase 1 and phase 2. All the cases were created according to different emotional tone and urgency to evaluate the flexibility of the model and the appropriateness of the responses.

The ratings were done in each interaction according to a five-point rubric that evaluated the following dimensions in Table 4.

**Table 4:** Evaluation rubrics for Sub-Agent response quality.

Criterion	Description	Scale (1–5)
Helpfulness	Extent to which the response provided meaningful guidance or actionable support	1 = Unhelpful, 5 = Highly helpful
Empathy	Ability to acknowledge emotional context and respond sensitively	1 = Insensitive, 5 = Highly empathetic
Clarity of Instruction	Degree to which the advice was concrete, understandable, and contextually appropriate	1 = Vague, 5 = Highly clear
Tone appropriateness	Suitability of language and emotional tone to the user's situation	1 = inappropriate, 5 = Highly appropriate
Consistency with policy	Alignment of responses with institutional guidelines and escalation procedures	1 = Inconsistent, 5 = Fully consistent

To compare model performance, average scores of all evaluators were compared.

#### 4.2.2 Quantitative findings - Phase 1

Phase 1 assessment took into account a range of Sub-Agents models, such as Gemini 2.5 Pro, Gemini 2.5 Flash, Gemini 2.5 Flash-lite, Gemini 2.0 Flash, and Gemini 2.0 Flash-lite with the results summarized in Table 5. Gemini 2.5 Pro scored the best in all qualitative areas, showing good guidance, understanding responses, and policy compliance, but with the most time to respond at 50seconds, it had limited practical real-time applications. Gemini 2.5 Flash and 2.5 Flash-lite offered a little less guidance quality but had response time of 20 seconds and 7 seconds respectively, which is more appropriate when it comes to interactive support. Although also fast, Gemini 2.0 Flash and Gemini 2.0 Flash-lite produced more generic responses and less sensitive to emotion and inconsistency in policy adherence. In general, Phase 1 underscored the existing performance, and areas where changes were necessary in context sensitivity, emotional continuity and proportional escalation were established, which led to the development of Phase 2 using AGE framework, Knowledge base and chain of thought prompting method.

**Table 5:** Comparative performance metrics of Sub-Agent models in Phase 1 evaluation.

Model	Avg. helpfulness score	Avg. empathy score	Avg. clarity score	Avg. tone appropriateness	Avg. consistency with policy	Avg. response time (s)
Gemini 2.5 Pro	4.8	5.0	4.7	4.9	4.8	50
Gemini 2.5 Flash	4.2	4.3	4.1	4.2	4.1	20
Gemini 2.5 Flash-lite	4.1	4.2	4.1	4.1	4.1	7
Gemini 2.0 Flash	3.9	4.0	3.8	4.0	4.0	7
Gemini 2.0 Flash-lite	3.7	3.9	3.6	3.8	3.9	6.5

#### 4.2.3 Qualitative insights—Phase 1

Qualitative analysis made a number of significant conclusions about Sub-Agent performance during Phase 1. Gemini 2.5 Pro model was also generally highly empathetic and contextually sensitive but had a high level of adherence to the policy and the length of response reduced the usefulness in real time interactions. The Gemini 2.5 Flash model was moderate in its empathy and level of guidance, though it sometimes gave generic reassurance instead of advice that was situation-specific. There were additional restrictions in flash-lite and Gemini 2.0 versions such as reduced continuity in emotions, sudden jump between guidance and reassurance, and slight variation in escalation cues. All these findings point to the necessity of systematic prompting, situational argumentation, and the use of structured models like A.G.E. as a means of ensuring the high level of support interaction with the client, which is emotionally engaging.

#### 4.2.4 Quantitative findings—Phase 2

Table 6 summarizes the outcomes of both tested models that were carried out in Phase 2, Gemini 2.5 Flash, and Gemini 2.5 Flash-lite. The two models were found in Phase 2 evaluation since they provided the optimum compromise of the quality of guidance and the response time in the Phase 1, surpassing other variants in the scores as well as efficiency. Phase 2 involved the use of the A.G.E. framework, structured knowledge base (policy documents) and chain-of-thought prompting, which further promoted context sensitivity, emotional continuity, and actionable advice during interactions with users.

**Table 6:** Comparative performance metrics of Gemini 2.5 Flash and Flash-lite Sub-Agent models.

Model	Avg. helpfulness score	Avg. empathy score	Avg. clarity score	Avg. tone appropriateness	Avg. consistency with policy	Avg. response time (s)
Gemini 2.5 Flash	4.6	4.8	4.5	4.7	4.6	25
Gemini 2.5 Flash-lite	4.3	4.4	4.2	4.3	4.1	20

**Figure 8:**
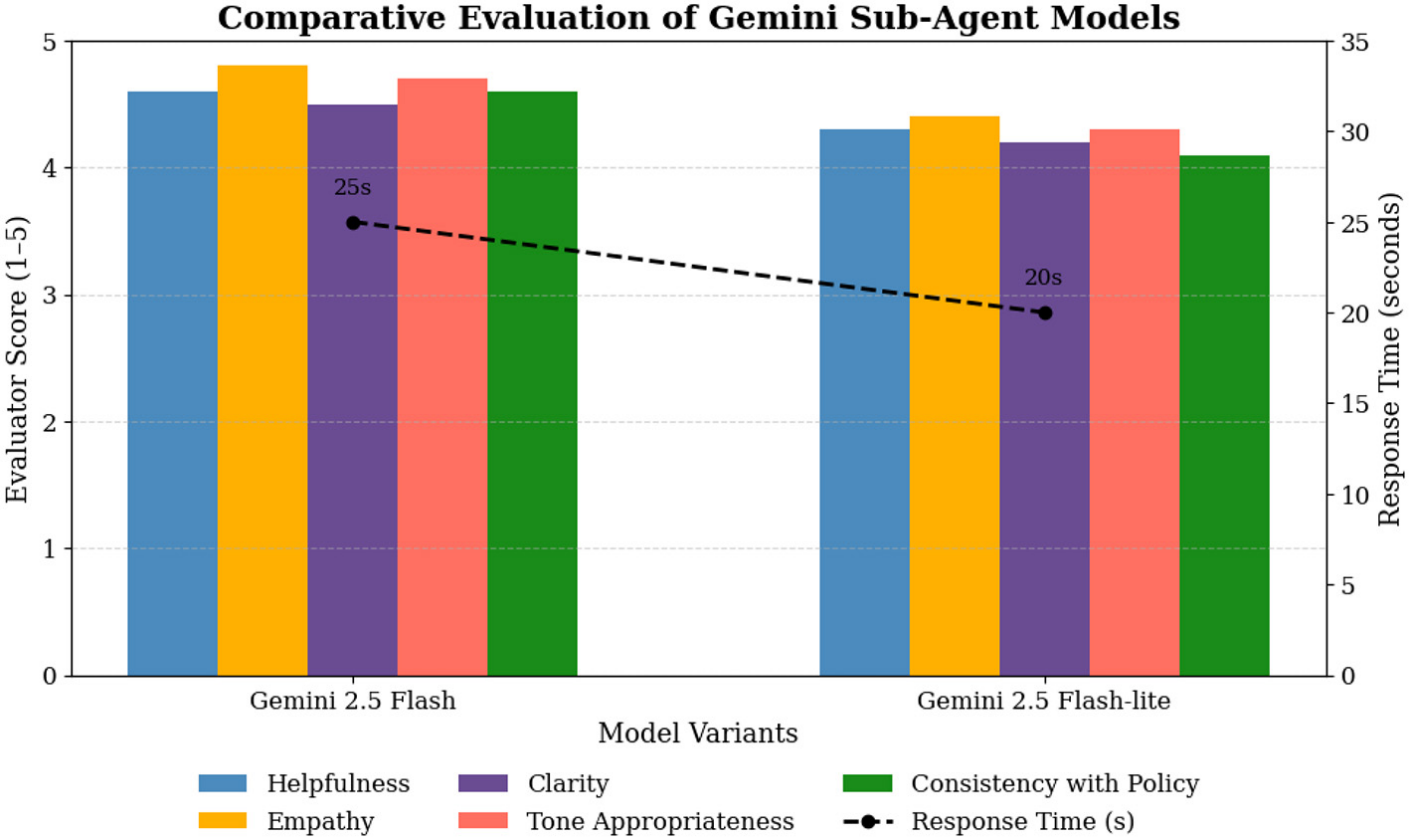
Comparative evaluation of Sub-Agent guidance quality across different criteria.

As shown in Figure 8, the Gemini 2.5 Flash model always exhibited high results in comparison to Flash-lite in all qualitative aspects. It generated more natural, contextually sensitive and emotionally sensitive responses. Its greater contextual continuity, especially that of the capability to retain awareness of user tone and sentiment through a series of turns in dialogue, increased perceived reassurance.

The Flash-lite model on the other hand, although faster in response generation did show some small conversational discontinuities or sudden transitions between empathy and practical advice. However, it is less latent and hence it is suitable in large scale real-time triage systems.

#### 4.2.5 Qualitative insights—Phase 2

The qualitative analysis provided a number of important conclusions regarding the performance of the Sub-Agent. The Gemini 2.5 Flash model was very consistent in empathy as it showed continuity in emotions throughout the multi-turn conversation and thus gave the users confidence.

The integration of the A.G.E. framework (Acknowledge-Guide-Escalate) also helped to enhance the response structure by substituting the general reassurance with situational and practical advice. Besides, the revised Sub-Agent exhibited more proportional escalation than earlier models, which were able to provide a proper recommendation based on the seriousness of each situation. Contrarily, the Flash-lite version had a few minor constraints such as redundant reassurance messages and a little less accurate escalation prompts. In general, these results emphasize the role of systematic prompting and potential maintenance of a conversational situation in high-quality, emotionally connective support interactions.

#### 4.2.6 Summary

Comparing Phase 1 and Phase 2 shows significant performance of Sub-Agents. Phase 1 set a moderate to high standard in terms of guidance quality, emotional sensitivity and policy compliance although response times were widely varying with Gemini 2.5 Pro being the fastest but too slow to be effective and Flash, Flash-lite, and version 2.0 were quicker but less sensitive to context. The application of A.G.E. framework and systematic prompting during phase 2 improved empathy, contextual continuity, and proportional escalation considerably. Gemini 2.5 Flash in Phase 2 had highest ratio of the quality of guidance and response time that offered natural, emotionally consistent and policy-oriented support that could be used in real-time in educational-institute applications. On the whole, the findings indicate the actual value of the structured prompting and framework-directed reasoning in comparison with the generic LLM output.

### 4.3 Pillar 2 and Pillar 3 evaluation

#### 4.3.1 Pillar 2: human escalation and support

Pillar 2 was created as a means to have a non-formal guiding system, discreet, and human-centric approach to cases in which the AI-assistance was seen as inadequate or the user needed the personal attention of the human. In evaluation, 60 controlled test scenarios were run in various categories of harm including harassment, ragging, property damage and wellbeing in general. Among them, 21 cases were transferred to human-level support via the special escalation system.

At the moment of escalation, the sub-agent engaged in a short reflective evaluation of the emotional resilience and coping capacity of the user, and produced a score on courage using a 5-point scale. High, moderate, and low priorities were automatically given to cases with a score of less than 2.0, between 2.1 and 3.5, and cases with a score of more than 3.5 respectively. This scoring system facilitated the opportunity of having a structured triage process which enabled the system to identify urgency and provide human attention promptly where necessary.

**Figure 9:**
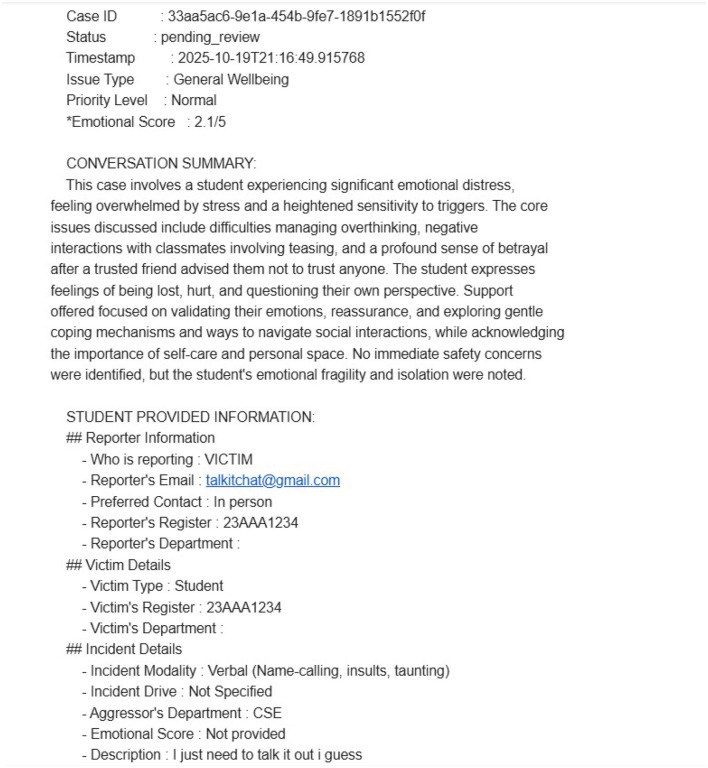
Escalation information sent to authorities.

**Figure 10:**
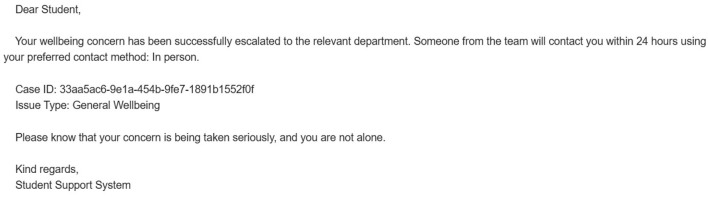
Acknowledgment message sent to user.

After the assessment, the users were asked to provide contextual information, including the incident modality, incident driver, the department of the aggressor and a short description of the incident. The information was requested only on the basis of what is required and the user had the option to share personal information on their own will. These details were then submitted and a structured escalation summary was generated and sent to the corresponding category-specific contact channels (e.g., different IDs were used to report harassment, ragging or wellbeing-related issues). The user was shown a confirmation message stating that he or she successfully escalated and a case identifier was given. An example of such acknowledgment message and the organized escalation information that was documented during evaluation can be seen in Figures 9, 10.

Security, privacy and confidentiality were design considerations throughout the workflow of escalation. Only real escalation and evaluation purposes were obtained using the data collected and transferred to the secure channels and stored within the controlled research boundaries. There were rigorous access control measures such that, only the authorized evaluators had the right to access sensitive records. The data protection principles of anonymization, encryption, and controlled data manipulation were adhered to and ensured confidentiality. In future analytics in Pillar 3, the anonymity was strictly enforced no personally identifiable information (PII) was stored or associated with analytical outputs. This holistic security design was able to ensure the trust of the users and provide the means of reliable end-to-end verification of the human escalation and institutional analytics procedure without undermining the ethical and privacy principles.

#### 4.3.2 Pillar 3: prevention and analytical insight

Pillar 3 expands the system's scope to institutional analytics and preventive intelligence. This pillar consolidates anonymized and aggregated information from both Pillar 1 and Pillar 2 to discover underlying trends, while preserving user privacy and confidentiality. All data used in Pillar 3 is anonymized, encrypted, and stored securely, maintaining confidentiality and protecting sensitive information.

**Figure 11:**
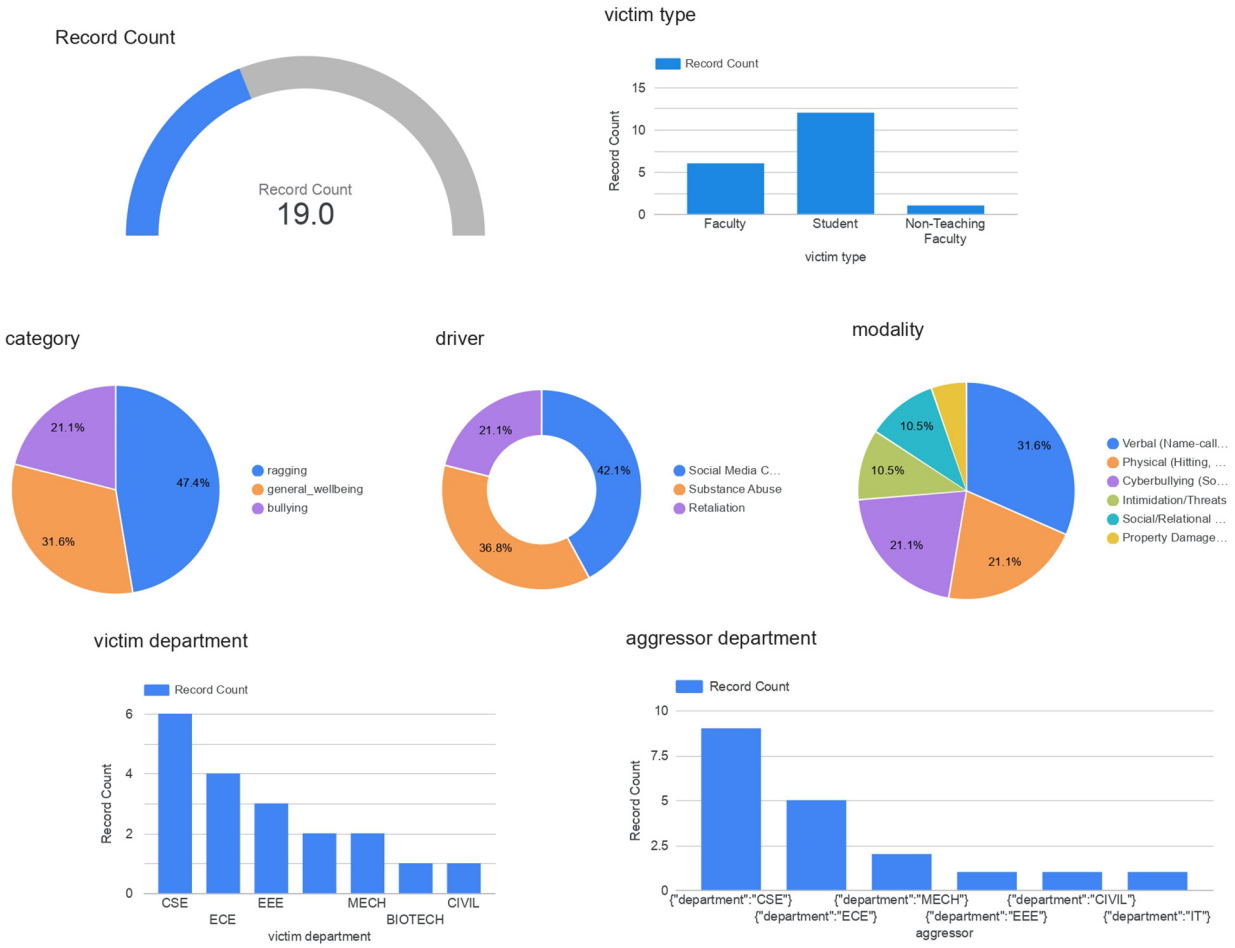
Analytics—looker studio dashboard.

The analytical dashboard (as shown in Figure 11) suggested a coherent view of wellbeing indicators by different dimensions such as the number of records, type of harm, classification of victim (student, faculty, or non-teaching staff) and drivers of incidences, the department of the victim, and the department of the aggressor. Such visualizations allowed highlighting such issues as recurrence, cross-departmental trends, and the new risk clusters.

Pillar 3, through information on structured escalation, was able to portray how insights could be employed to shape preventive measures, including awareness efforts, counseling interventions, and focused welfare programs. The framework will guarantee that although the institutional visibility is established on the macro level, the individual confidentiality is not compromised, in accordance with the data ethics and responsible AI principles.

The combination of Pillar 1, 2, and Pillar 3 indicates the capability of the system to transition to a proactive prevention, as opposed to immediate response, and creates a continuous and ethically-grounded cycle of detection, escalation, and institutional response.

## 5 Discussion

The two-phase test of the SocialWellbeing System discovered significant information about difficulties and possibilities of using large language models (LLMs) in sensitive and high-stakes contexts such as psychosocial support. While an initial stage that relied on prompt engineering using few-shot examples revealed weaknesses in classification accuracy, response quality, and latency (Liu et al., 2023), a subsequent stage that used a formalized chain-of-thought prompting design based on the A.G.E. (Acknowledge–Guide–Escalate) framework (Wei et al., 2022) produced impressive improvements. Nonetheless, the improvements also uncovered an alarming failure mode, which we call the Proportionality Paradox. The results obtained are interpreted below in three subsections. First, we argue that general-purpose, non-guarded LLMs cannot be used in high-stakes applications (Gabriel, 2020). Second, we present the Proportionality Paradox as a new problem in AI safety, which brings sensitivity and proportionality into conflict. Third, we reflect on how the in-built mechanism provides an example of institutional change through the integration of AI support, human intervention, and preventive measures (Andersson et al., 2019).

### 5.1 Beyond general models: the need for engineered guardrails

Applying this technology to real-world situations initially revealed problems: the prompt-engineered model with few-shot examples missed nuanced dangers and sometimes offered questionable advice (Weidinger et al., 2021). In response, the follow-up stage introduced targeted interventions—most notably a “Master Agent” instruction to improve categorization and the A.G.E. (Acknowledge–Guide–Escalate) system to improve responses. The A.G.E. method listens to users, offers help, then seeks human assistance when required. Kindness and empathy matter, but AI also needs clear, well-designed boundaries to handle delicate issues responsibly (Gabriel, 2020). These engineered guardrails align system behavior with core values and produce more secure, predictable exchanges.

### 5.2 The proportionality paradox: a new frontier in AI safety

The A.G.E.-directed framework reduced a number of the limitations that were found in the previous stages of evaluation, including increased empathy, contextual continuity, and proportional escalation as shown in Table 5. However, it also created a new problem the Proportionality Paradox. Increased sensitivity of the system although good in detecting serious cases like bullying and harassment, sometimes led to over-blown conditions. For instance, user statements that contain implicit protective sentiments expressing subtle feeling of exclusion that have been inappropriately labeled as bullying, resulting in the disproportionate responses, including security warnings. However, as much as these reactions showed high protection intent, they were too vigorous enough to compromise user trust and psychological comfort. In the context of psychosocial support, these overreactions may be just as harmful as inadequate responses, which may make users feel discouraged to continue engaging in the system. Therefore, proportionality of response mechanisms is necessary to ensure the establishment of a psychologically safe interaction environment and support the ongoing disclosure of users (Inkster et al., 2018).

Usually, concerns focus on missed dangers, but overreaction has its own harms. A more balanced approach is to start with gentle, de-escalatory support for ambiguous signals—for example: “It sounds like you're feeling a bit left out. Would you like to talk more or see some tips?”—and request more detail before escalating. This keeps the interaction calm and supportive without jumping to extremes. Systems should explicitly represent uncertainty and prefer proportionate, context-sensitive steps: quick empathic prompts for minor issues, deeper exploration for unclear cases, and rapid escalation only when indicators cross clearly defined thresholds (Wang et al., 2021).

### 5.3 Integrated system as a model for institutional change

Three interconnected principles show how well-structured AI can positively address psychosocial problems in schools, universities, workplaces, and other organizations via the SocialWellbeing System (Andersson et al., 2019):

Immediate, compassionate assistance at scale. Purpose-driven AI can act as an always-available, non-judgmental first contact that encourages reporting. This is important because many individuals are reluctant to report harassment or distress—fearing loss of confidentiality or dismissal of their concerns (Hugh-Jones et al., 2022). A gentle AI can lower that barrier and encourage users to seek help.

Facilitating, not replacing, human care. The AI is not a replacement for human interaction; it is a facilitator. It routes users to appropriate counselors, peers, and formal support, making the handoff from digital to human care seamless and instinctive. Combining technology and human resources is frequently the most successful strategy, according to research on blended mental health care (Andersson et al., 2019).

By collecting anonymized data, institutions can identify systemic problems (like ongoing stress or cycle harassment) and implement preventative strategies like awareness campaigns or policy changes (Wies et al., 2021). Organizations frequently ignore these trends due to a lack of timely, aggregated data; the system helps highlight those areas so that prevention, not just response, is feasible.

These ideas demonstrate that artificial intelligence (AI) is more than just a technical tool; with the right design, it could improve institutions' and people's policies and services (Gabriel, 2020). The first fewshot prompt stage can be replaced with more formal chain-of-thought designs (like A.G.E.) to increase safety and usefulness. However, systems must also contain proportionality and uncertainty to prevent problems like the Proportionality Paradox.

## 6 Limitations

This study is exploratory and scenario based. It used 60 anonymized, student authored fictionalized vignettes rather than live user disclosures. Scenario labeling was conducted by a five person panel composed entirely of the study authors (three student co-authors, an industry expert, and the supervising research professor); initial labels were produced independently and final consensus labels were determined during an adjudication meeting between the industry expert and the supervising research professor. Individual independent rater logs were not archived. Because labellers included scenario authors and core study team members, and because the dataset is synthetic and modest in size, the findings may be subject to labeller bias and have limited generalizability. Future work should validate these results using independent, blinded raters, larger and more diverse scenario sets, and real world pilot deployments. The other limitation is that of external model dependency. Though the behavior was observed to be stable over the evaluation time-span, the dependency on an external LLM API creates a risk of theoretical dependency, wherein, the performance of the upstream model is updated or the policy, in extreme instances, may influence the performance consistency.

## 7 Conclusion

We looked at the process of creating an AI system to promote personal wellness, a project that required constant improvement and careful consideration. This simulation-based exploratory study suggests that systematic testing, error detection, and iterative performance assessment can be used to enhance the ability of the system to handle delicate human interactions though additional confirmation on this point of view using real-life data is also required. It highlights how crucial thorough verification is when creating safety-critical and compassionate AI systems. We created the *Acknowledge–Guide–Escalate (A.G.E.)* framework, a structured response model intended to identify users' feelings, offer insightful guidance, and escalate when required, in order to better support users. This multi-layered strategy guarantees that users are protected by clear escalation procedures, feel understood, and receive helpful guidance.

In the end, this framework preserves system dependability and ethical safety while fostering user trust. A recurrent problem surfaced: the AI occasionally overreacted to minor problems because it was too careful to prevent serious harm. This exemplifies the fine line that separates proportionality from responsiveness. Being overly sensitive can undermine user confidence or cause needless worry, even though being vigilant is necessary to prevent missing important situations. The key to developing reliable support-oriented AI is still striking a balance between attentiveness and restraint. The study had limitations even though it produced encouraging results. The generalizability of the evaluation results' is still unknown because it was conducted in simulated rather than real-world settings.

Future research ought to test the system in real-world learning or work settings in order to completely evaluate its efficacy. Such implementation would provide more in-depth understanding of how adaptive balancing, which involves dynamically modifying responses in response to changing context and user input, can improve overall accuracy and emotional intelligence. Building AI that actually benefits people is ultimately important from a societal standpoint. Although tested in controlled conditions with a relatively small synthetic dataset, the findings indicate that properly crafted AI solutions can assist in education, wellness and self-development at work, and that the evaluation process and testing in the real environment can improve and prove their efficiency. We're also just at the beginning of the generative AI era, with version 1.0 of a rapidly evolving field. The prospect of dependable, context-sensitive AI is going to increase rapidly over the next few years, as new LLMs with enhanced reasoning, reduced latency, and sophisticated orchestration features like MCPs are released every few months. While the *Gemini 2.5 Pro* model used in our experiments showed remarkable empathy and reasoning, latency was still a constraining factor. This limitation should rapidly diminish as newer LLMs with faster response times and sophisticated agent coordination become available. Based on these advancements, the A.G.E. framework can evolve into a more robust hybrid ecosystem incorporating many specialized agents, as described in Section 3.3.2, to further enhance scalability, responsiveness, and reliability. The combination of technical proficiency and moral delicacy seen in simulated testing is a positive move toward anthropocentric AI systems, and further refinement and testing in larger and real-world scale is necessary to improve and prove this method.

These findings are practice informed and exploratory. We present the *A.G.E. framework* and discuss the 'proportionality paradox' as conceptual contributions to guide further research and deployment considerations for psychosocial AI. Further validation with independent raters and field studies under formal institutional ethics approval is required prior to deployment.

## Data Availability

The synthesized data supporting the conclusions of this article will be made available by the authors without undue reservation.
